# Endothelial Function in Healthy Young Individuals Is Associated with Dietary Consumption of Saturated Fat

**DOI:** 10.3389/fphys.2017.00876

**Published:** 2017-11-09

**Authors:** Elisabeth A. Lambert, Sarah Phillips, Regina Belski, Ainura Tursunalieva, Nina Eikelis, Carolina I. Sari, John B. Dixon, Nora Straznicky, Mariee Grima, Geoffrey A. Head, Markus Schlaich, Gavin W. Lambert

**Affiliations:** ^1^Faculty of Health, Arts and Design, Iverson Health Innovation Research Institute, Swinburne University of Technology, Hawthorn, VIC, Australia; ^2^Human Neurotransmitters Laboratory, Baker Heart and Diabetes Institute, Melbourne, VIC, Australia; ^3^Department of Health Professions, School of Health Science, Swinburne University of Technology, Hawthorn, VIC, Australia; ^4^Department of Statistics Data Science and Epidemiology, School of Health Science, Swinburne University of Technology, Hawthorn, VIC, Australia; ^5^Clinical Obesity Research Laboratory, Baker Heart and Diabetes Institute, Melbourne, VIC, Australia; ^6^Department of General Practice, Monash University, Clayton, VIC, Australia; ^7^Neuropharmacology Laboratory, Baker Heart and Diabetes Institute, Melbourne, VIC, Australia; ^8^Dobney Hypertension Centre, School of Medicine—Royal Perth Hospital Unit, University of Western Australia, Perth, WA, Australia

**Keywords:** diet, saturated fat, endothelial function, overweight, cardiovascular risk

## Abstract

**Background:** A diet rich in fat, in particular saturated fat (SF), may be linked to cardiovascular disease development, possibly due to a detrimental effect of fat on endothelial function (EF).

**Objective:** We aimed to determine whether the habitual SF intake [as a ratio to total fat (the sum of saturated, polyunsaturated, and monounsaturated fat)] might influence endothelial function in young, overweight but otherwise healthy adults.

**Design:** Sixty-nine young adults (49 males, mean age: 23 ± 1 years, mean BMI: 29.1 ± 0.8 kg/m^2^) were classified into three tertiles according to their habitual SF intake consumption (low SF: <39%, medium SF 39.1–43.7%, and high SF: >43.7% of total fat). Endothelial function was assessed using digital amplitude tonometry.

**Results:** The three groups of individuals were comparable for total energy intake and calories from: fat, protein, and carbohydrates. There was no difference in anthropometric and hemodynamic variables among the groups. Those in the high SF group presented with impaired endothelial function [reactive hyperemia index (RHI): high SF: 1.60 ± 0.08 compared to 2.23 ± 0.16 in the medium SF and 2.12 ± 0.14 in the low SF group, *P* < 0.01]. Regression analysis, including gender, age, ethnicity, body mass index indicated that the ratio of SF to total fat was an independent predictor of the RHI (*P* < 0.05).

**Conclusion:** The habitual consumption of a diet high in SF in relation to polyunsaturated and monounsaturated fat was strongly associated with impaired endothelial function in young overweight adults, potentially contributing to increased risk of developing cardiovascular disease.

## Introduction

Cardiovascular diseases (CVD) remain the major cause of death in developed and developing countries. Lifestyle factors such as lack of physical activity and poor diet are likely to play an important role in the development and progression of CVD. It has long been believed that total saturated fat (SF) intake was associated with an increased risk of coronary heart disease (CHD) or CVD. However, a recent meta-analysis of prospective epidemiological studies indicated that there was not significant evidence for concluding that total dietary SF intake is associated with an increased risk of CHD or CVD (Siri-Tarino et al., 2010). Another recent systematic review of 607 studies examining the effect of the amount and type of dietary fat on cardiometabolic risk factors concluded that there was convincing evidence that partial replacement of SF with polyunsaturated fat decreases the risk of CVD, especially in men, and that partial replacement of SF with polyunsaturated or monounsaturated fat lowered fasting serum/plasma total and LDL cholesterol concentrations (Schwab et al., 2014) suggesting that the ratio of saturated fat to total/unsaturated fat may be more important than total intake.

While many clinical and experimental studies have demonstrated that increased consumption of fat can lead to excess weight (Moussavi et al., 2008), dietary fat may not necessarily be a determinant of body fat (Willett and Leibel, 2002). Nevertheless, overweight and obesity can be regarded as risk factors for CVD (Mandviwala et al., 2016). Aside from a possible effect on adiposity, diets high in SF have been shown to exert detrimental effects on plasma LDL cholesterol levels, inflammation, insulin sensitivity, and atherogenesis which, together promote the development of metabolic abnormalities and cardiometabolic disease (Brunner et al., 2001). Endothelial dysfunction is recognized as a critical, early, modifiable event in the development of coronary and general atherosclerosis and is strongly associated with increased CVD risk (Hadi et al., 2005). Endothelial dysfunction is also commonly seen in overweight individuals, even at an early age and prior to the clinical manifestation of CVD (Singhal, 2005). Recently it was found that adults consuming a regular diet high in fat, especially in SF, presented with lower endothelial fibrinolytic function, a marker of increased atherothrombic disease risk (Dow et al., 2014) and that in young healthy males, endothelial vasodilatory function was inversely related to the proportion of plasma concentration of saturated fatty acid and positively related to the plasma level of alpha-linolenic acid, an unsaturated fatty acid (Steer et al., 2003).

Whether the quality of fat as opposed to the quantity of fat regularly consumed impacts on early signs of CVD risk is not clear. Some investigators have proposed that the quality of dietary fat and not just the quantity may be more closely related to the risk of developing cardiovascular complications (Riccardi et al., 2004) and this is supported by the findings of the review by Schwab et al. (2014). As endothelial dysfunction is an early marker of CVD development and is linked with the development of obesity, the aim of the present study was to assess whether endothelial function was associated with the quality of dietary fat intake, as indicated by the ratio of saturated fat to total fat, in a group of young, predominantly overweight and class I obese, but otherwise healthy adults.

## Methods

The data was extracted from the Human Neurotransmitters Laboratory database of the Baker Heart and Diabetes Institute, which comprised subjects who had participated in relevant studies conducted between 2008 and 2016. They fulfilled the following criteria: aged between 18 and 30 years, healthy, non-smokers, not on any medication, and not taking any over the counter medications or supplements, and for whom dietary records and measures of endothelial function were obtained. None of the participants had a history of cardiovascular, metabolic or cerebrovascular disease. All participants underwent a clinical examination. The Alfred Hospital Human Ethics Committee approved the study protocol and all participants gave written informed consent before participating in the study.

Participants attended the clinic/center at 0900 h having fasted for 12 h and abstained from caffeine for at least 18 h and from alcohol for at least 36 h.

Demographic details including age, gender, race, and blood pressure (BP) were obtained from standard measurements and questionnaires. Participants reported their habitual physical activity as time per week spent in moderate and vigorous intensity physical activity. Supine BP was measured three times after 5 min rest using a Dinamap monitor (Model 1846SX, Critikon Inc, Tampa, FL, USA) and values were averaged. Body weight was measured in light indoor clothes without shoes using a digital scale. Waist circumference was measured at the midpoint between the lowest rib and iliac crest, and hip circumference at the level of the greater trochanters.

Venous blood was drawn from a cannula placed in an antecubital vein for the measurement of the metabolic variables including lipids, glucose, insulin, and C-reactive protein (CRP). These standard measurements were performed by the pathology department of the Alfred Hospital. Insulin resistance index (HOMA-IR) was calculated according to the formula: fasting insulin (microU/L) x fasting glucose (nmol/L)/22.5.

Participants were instructed by a nutritionist (MG) on how to record their dietary intake for 4 days (3 consecutive weekdays and 1weekend day) and not to alter their regular diet during the study period. Throughout this period participants were to record the exact items, brands, and quantities of foods consumed. If the food was prepared from various ingredients, participants were required to provide the recipe. Upon completion, these food diaries were analyzed using the Australian Food Composition tables of the FoodWorks® Professional dietary analysis software (Version 3.02, Xyris Software, Highgate Hill, Australia) based on the Australian food composition tables (Cashel et al., 1989). The resulting reports gave the participants total energy intake, as well as values for macro-nutrients (e.g., fat, carbohydrate, protein) as well as micro-nutrient consumption.

### Digital vascular function

Endothelial function was measured with a pulse amplitude tonometry (PAT) device placed on the tip of each index finger (Itamar Medical Ltd., Caesarea, Israel). Pulse amplitude tonometry (PAT) was assessed in response to reactive hyperemia. Measurements were obtained for 5–10 min at baseline followed by 5 min of occlusion of one arm, with the cuff inflated on the upper arm to supra-systolic pressure (60 mmHg above systolic pressure or 200 mmHg) and then released to induce reactive flow-mediated hyperemia, measured for 5–10 min. Reactive hyperemia index (RHI) was calculated as the index of signal amplitude pre-to-post occlusion in the occluded arm, divided by the same ratio in the control arm. Previous studies have demonstrated good correlation of repeated digital EF measurements using PAT technology performed on different days in healthy volunteers (McCrea et al., 2012), and this method has been validated against flow mediated dilation (Kuvin et al., 2003).

## Data analysis

The ratio of saturated fat/total fat (sum of saturated, polyunsaturated, and monounsaturated fats) was calculated and used to divide the participants into tertiles. This classification was used to characterize the physiological parameters according to the quality of fat intake (favoring saturated fat to the detriment of other fat) rather than the quantity.

Tables 1, 2 present data as mean ± SEMs. It was first determined if the means of variables reported in Tables 1, 2 were different among the tertile groups. Both normality and equality of variances assumptions were violated for almost every variable, therefore three groups comparison analysis was performed using Dunn's test with Bonferroni correction.

**Table 1:** Participant characteristics.

	**Low SF (*n* = 24)**	**Medium SF (*n* = 21)**	**High SF (*n* = 24)**
Men/Women	18/6	15/6	16/8
Age (years)	23 ± 1	23 ± 1	23 ± 1
Ethnicity (Asian/Caucasian/other)	14/9/1	10/9/2	12/12/0
Body mass (kg/m^2^)	28.8 ± 1.3	30.7 ± 1.5	28.1 ± 1.2
Waist circumference (cm)	92.3 ± 3.7	92.8 ± 3.4	88.7 ± 2.9
Physical activity (min/week)	189 ± 32	230 ± 38	192 ± 25
Systolic BP (mmHg)	117 ± 3	121 ± 3	118 ± 3
Diastolic BP (mmHg)	68 ± 2	68 ± 2	69 ± 2
Total cholesterol (mmol/L)	4.34 ± 0.13	4.57 ± 0.17	4.81 ± 0.15
LDL cholesterol (mmol/L)	2.59 ± 0.10	2.84 ± 0.14	2.95 ± 0.13
HDL cholesterol (mmol/L)	1.18 ± 0.06	1.20 ± 0.06	1.33 ± 0.05^*^
Triglycerides (mmol/L)	1.27 ± 0.16	1.16 ± 0.14	1.15 ± 0.11
Glucose (mmol/L)	4.62 ± 0.09	4.52 ± 0.11	4.55 ± 0.07
Insulin (mU/L)	17.9 ± 2.0	16.2 ± 1.2	16.5 ± 1.5
HOMA	3.77 ± 0.47	3.28 ± 0.29	3.30 ± 0.29
CRP (mg/L)	1.61 ± 0.47	1.90 ± 0.37	2.59 ± 0.45

*All values are means ± SEMs*.

High SF vs. low SF:

* *P < 0.05*.

**Table 2:** Dietary composition in the three groups of subjects defined as tertiles of Saturated fat, % of total fat.

	**Low SF (*n* = 24)**	**Medium SF (*n* = 21)**	**High SF (*n* = 24)**
Total Energy, kilojoules	9676 ± 589	9689 ± 785	9531 ± 621
Carbohydrates, % of total energy	46 ± 2	43 ± 1	46 ± 1
Protein, % of total energy	19 ± 1	20 ± 1	17 ± 1
Fat, % of total energy	33 ± 1	35 ± 1	34 ± 1
Saturated fat, % of total fat	36 ± 1	42 ± 0^‡‡‡^	47 ± 1^***^,^†††^
Monounsaturated fat, % of total fat	44 ± 1	41 ± 0^‡^	39 ± 1^***^,^†^
Polyunsaturated fat, % of total fat	20 ± 1	17 ± 1	14 ± 0^***^,^†††^
Total Fat, g/day	85 ± 5	92 ± 9	90 ± 8
Saturated fat, g/day	28 ± 2	35 ± 4	39 ± 4^**^
Monounsaturated fat, g/day	34 ± 2	35 ± 3	32 ± 3
Polyunsaturated fat, g/day	15 ± 1	14 ± 1	11 ± 1^**^

*All values are means ± SEMs*.

High SF vs. low SF:

** *P < 0.01*,

*** *P < 0.001*.

High SF vs. medium SF:

† *P < 0.05*,

††† *P < 0.001*.

Medium SF vs. low SF:

‡ *P < 0.05*,

‡‡‡ *P < 0.001*.

The relationship of RHI with SF, monounsaturated fat, polyunsaturated fat, and the relevant hemodynamic variables was estimated using multiple linear regression with RHI as a dependent variable. The Spearman's correlation coefficients (CC) were used to select variables associated with RHI. The variables with *p*-values of less than 0.1 were included in the regression. Furthermore, the following demographic and anthropometric variables were included: gender, age, ethnicity, and BMI. A variable with the *p*-value for the *t*-test of less than 0.05 was considered to have a statistically significant linear relationship with RHI.

All analysis was performed with R version 3.3.3 (2017-03-06).

## Results

A complete set of data was available in 69 participants (49 males). The ethnicity background was: Asian: 36, Europid: 30, African: 1, Middle Eastern: 1, South American: 1. Participants were 23.0 ± 0.4 years old and had a BMI of 29.1 ± 0.8 kg/m^2^ (Table 1). Fifty-six individuals (81%) were classified as either overweight or obese (BMI > 25 kg/m^2^).

The three groups were defined as tertile 1: low % of saturated fat intake (low SF: ≤39.3%, *n* = 24), tertile 2: medium % of saturated fat (medium SF: >39.3% to ≤43.7%, *n* = 21), and high % of SF (high SF >43.7%, *n* = 24).

Table 2 shows dietary macronutrient composition of the groups. The groups were similar for total energy intake. In addition, the percentage of energy from both carbohydrate, protein, and total fat did not differ between groups. As per group allocation, there was a graded increase in proportion of SF and decrease in monounsaturated and polyunsaturated fat intake across the low, medium, and high SF groups. While there was no difference among the groups with regards to the amount of total fat consumed (in g/day), the high SF group was characterized with higher consumption of SF and lower consumption of polyunsaturated fat.

There were no significant differences between the groups in any anthropometric or hemodynamic variables (Table 1). The metabolic variables were similar for fasting plasma glucose, insulin, HOMA, CRP, triglycerides, total cholesterol, and LDL-cholesterol. HDL cholesterol was slightly higher in the high SF group compared to the low SF group (*P* < 0.05).

**Figure 1:**
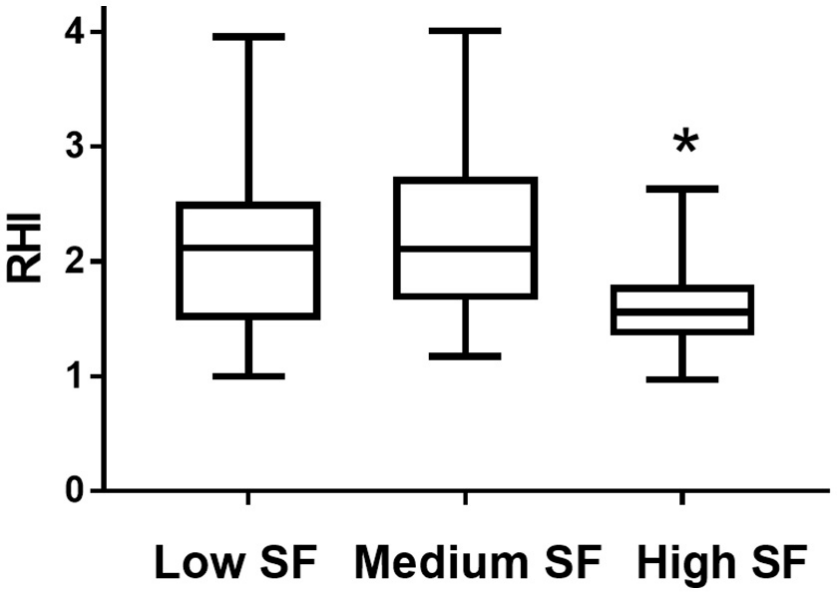
Reactive Hyperaemia index (RHI) in individuals with habitual consumption of saturated fat (percentage of saturated fat/total fat) in the lowest tertile (Low SF), second tertile (Medium SF), and highest tertile (high SF). Data is presented as mean with minimum and maximum values. ^*^Indicates *P* < 0.01 High SF vs. Low SF and High SF vs. Medium SF.

Endothelial function, as assessed from the RHI determination, was significantly lower in the high SF group compared to the medium SF and low SF groups (1.60 ± 0.08 [high SF] vs. 2.23 ± 0.16 [medium SF], *P* < 0.01 and vs. 2.12 ± 0.14 [low SF], *P* < 0.01) (Figure 1).

**Figure 2:**
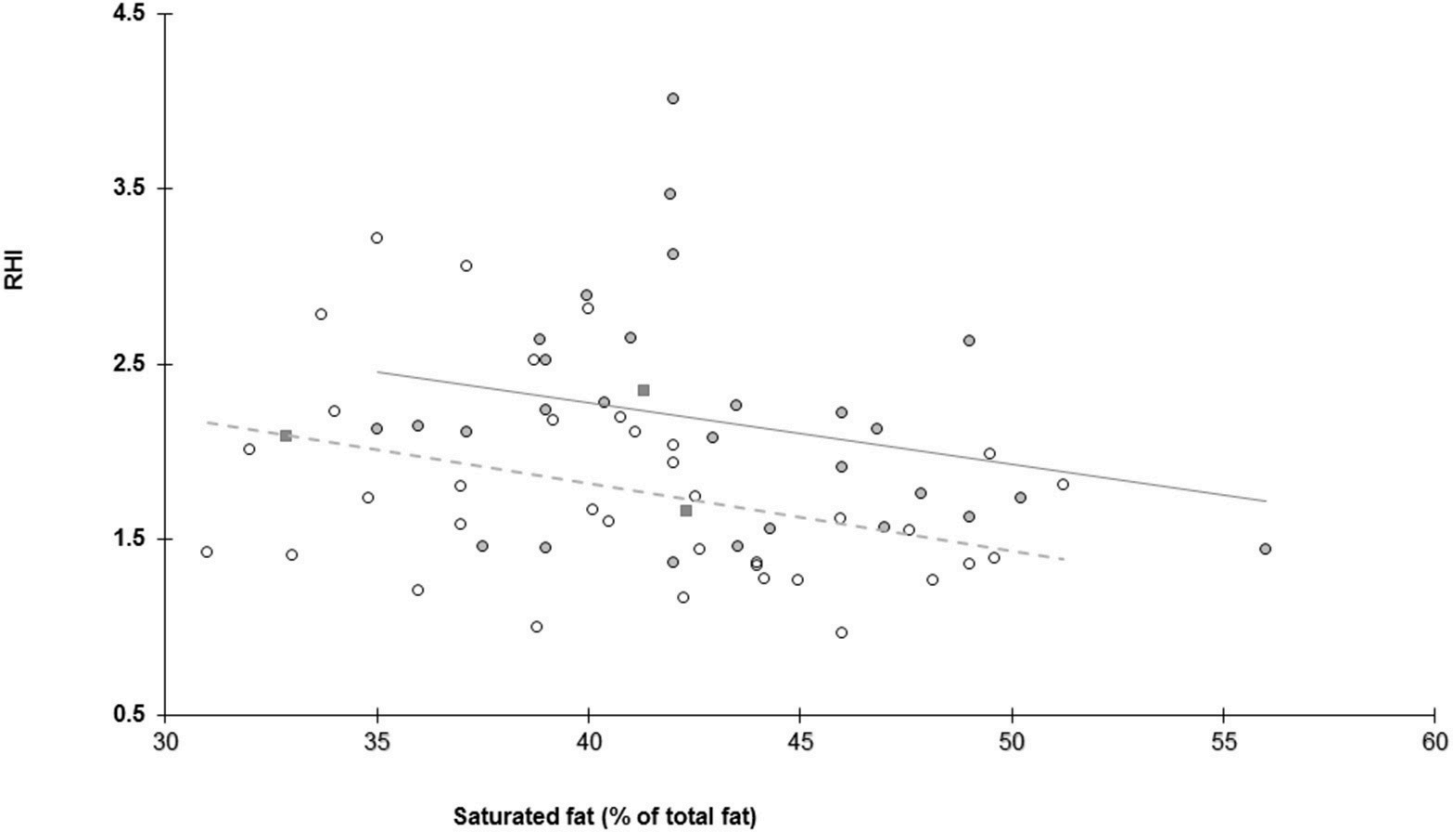
Relationship between endothelial function assessed as reactive hyperaemia index (RHI) and habitual consumption of saturated fat expressed as percentage of saturated fat/total fat. Dark circles and solid line represent individuals of European background, open circles and dashed line represent individuals with an Asian background and dark squares represent others.

Variables selected for multiple regression analysis were SF (% of total fat) (CC = −0.34, *P* < 0.001), glucose (CC = 0.24, *P* = 0.04), HDL cholesterol (CC = −0.29, *P* = 0.01), and total cholesterol (CC = −0.21, *P* = 0.08). Parameters not entered were: LDL (CC = −0.11, *P* = 0.37) triglycerides (CC = −0.05, *P* = 0.65), h-CRP (CC = −0.03, *P* = 0.81), systolic blood pressure (CC = 0.11, *P* = 0.38), and diastolic blood pressure (CC = 0.00, *P* = 1.00). Age, ethnicity, gender, and BMI were included. The % of SF was a significant negative predictor and European ethnicity a positive predictor of RHI (*R*^2^ = 0.17), explaining 17% of the variability (Table 3, Figure 2). Given that HDL and total cholesterol are related (Spearman's CC = 0.87, *P* = 0.15), regression analysis was also run entering either HDL or TC separately and this did not alter the results: When removing total cholesterol, HDL-cholesterol showed no association with RHI (*P* = 0.129). When removing HDL-cholesterol, total cholesterol showed no association with RHI (*P* = 0.327).

**Table 3:** Reactive hyperemia index (RHI): multivariate analysis *n* = 69.

**Independent variables**	**Estimated coefficient**	***t*-statistic**	***p*-value**
**Gender**			
Female (base)			
Male	−0.243	−1.234	0.222
**Ethnicity**			
Asian (base)			
Europid	0.438	2.720	0.009^**^
Other	0.091	0.214	0.832
Age, years	0.004	0.166	0.869
Body mass index (kg/m^2^)	−0.005	−0.323	0.748
Saturated fat, % of total fat	−0.036	−2.267	0.027^*^
Total cholesterol, mmol/L	−0.063	−0.548	0.586
HDL cholesterol, mmol/L	−0.440	−1.286	0.204
Glucose, mmol/L	0.346	1.792	0.078
Constant	2.625	1.903	0.062
*R*^2^	0.172		

* *P < 0.05*,

** *P < 0.01*.

## Discussion

Our study indicates that at a comparable level of daily energy intake and percentage of fat, carbohydrates, and protein, endothelial function was impaired in individuals who consumed a greater proportion of SF in relation to poly and mono-unsaturated fats. This observation suggests that the quality of fat regularly consumed may be an important determinant of endothelial function in a population of young overweight but otherwise healthy individuals.

Endothelial dysfunction, characterized by a reduction of the bioavailability of vasodilators, particularly nitric oxide, and/or an increase in endothelial-derived contracting factors, is thought to reflect the propensity to develop atherosclerosis, and thus may serve as a marker of an unfavorable cardiovascular prognosis (Hadi et al., 2005). We have previously demonstrated that endothelial function was impaired in young overweight individuals compared to those of normal weight (Lambert et al., 2010), as well as in young females with dyslipidemia (Lambert et al., 2013). In the present study, we demonstrated that dietary habits may influence endothelial function in young adults, with the ratio of SF to total fat being an important determinant of endothelial function, with the association being independent of weight and the plasma lipid profile.

The possible mechanisms underlying decreased endothelial function in individuals who favor the consumption of SF to other fat are not clear. Aside from fat quality, the groups were not different with regards to total energy intake and percentage of energy from fat, protein, or carbohydrate. The anthropometric, haemodynamic, and most metabolic parameters were not significantly different. Saturated fatty acids are recognized as the dietary factor that has the greatest negative effect on LDL cholesterol concentrations (Fernandez and West, 2005) and could thereby play an important role in the development of atherosclerosis (Tomkin and Owens, 2012). While we cannot exclude a role of plasma cholesterol, and specifically LDL cholesterol, we note that in our group of metabolically healthy young individuals, endothelial function was not related to total cholesterol levels and neither to LDL cholesterol concentrations. Ethnicity was a contributing factor influencing endothelial function with European background being associated with greater RHI. Differences in endothelial function between people of Asian and European background has been described in some (Yim et al., 2012) but not all studies (Pusalavidyasagar et al., 2016). Nevertheless, ethnicity did not alter the relationship between the consumption of SF and endothelial function.

Our observation that dietary habits, in particular fat quality may detrimentally influence endothelial function in healthy adults is in line with two previous studies demonstrating that endothelium-dependent vasodilation was impaired in individuals with a regular diet favoring high fat consumption (Dow et al., 2015), and that endothelial fibrinolytic dysfunction occurred in those consuming high levels of SF (Dow et al., 2014). The influence of diet quality on endothelial function has also been demonstrated in a number of intervention studies: Keogh et al. (2005) showed that in healthy individuals flow mediated dilation was reduced by 50% following consumption of a high SF diet for 3 weeks compared with a high-monounsaturated fat or polyunsaturated fat diet or a high carbohydrate diet, and this was independently associated with a rise in LDL cholesterol. Similarly, 4-week consumption of a Mediterranean diet (low in SF and high in monounsaturated fat) was associated with lower concentration of endothelial microparticles, a marker of endothelial injury, compared with consumption of other diets in healthy elderly participants (Marin et al., 2011).

Association studies have found that the consumption of SF is associated with higher BP (Stamler et al., 1997) and decreased insulin sensitivity (Riccardi et al., 2004). This was not apparent in our group of individuals, perhaps due to their young age and overall good health. In addition, while the vast majority of the participants in the present study were overweight, their daily energy intake and distribution of macronutrients were close to the recommended guidelines, albeit being in the upper range of recommended fat intake (2006)^1^ and their levels of physical activity were within the ranges recommended by current guidelines (2010)^2^.

Obesity and overweight is a growing health problem with its prevalence is increasing all over the world, particularly among young adults (Kelly et al., 2008). While the cause of this epidemic is certainly multifactorial, dietary factors remain a key aspect and as such first line treatment for obesity focuses on lifestyle factors including diet and exercise. Studies have shown either benefit (Pierce et al., 2008) or no effect (Mohler et al., 2013; Lambert et al., 2017) of diet-induced weight loss on endothelial function. The results of this study suggest that from a CVD risk reduction perspective, focusing on diet quality rather than weight loss *per se* may provide greater impact. For example, it was reported that in hypercholesterolaemic men, a diet low in fat, especially SF, and rich in monounsaturated fats improved endothelial function (Fuentes et al., 2001). Whether changing the proportion of SF in young adults who habitually consume a diet favoring SF to other fat would also improve endothelial function is not known. Of note, a recent study showed that introducing or replacing saturated and trans-fat with unsaturated fatty acids was beneficial in terms of cardiovascular risk reduction in obese or overweight non-diabetic elderly people due to improved endothelial markers of atherosclerotic disease (de Oliveira et al., 2017).

Limitations of the study include its cross-sectional nature and therefore no conclusion about causality can be drawn. Endothelial function was derived from the EndoPat technique, which uses pulse volume changes at the fingertips after an occlusion of the brachial artery. Although the method has been validated (Kuvin et al., 2003) it has a higher within-day variability compared to the more traditional method of flow mediated dilation (Onkelinx et al., 2012). Another limitation is that we relied on 4-day food records to assess energy intake, which potentially carries a self-reporting bias.

In conclusion, the present study indicates that a habitual dietary intake comprising a high proportion of SF at the expense of mono and poly-unsaturated fat is related to reduced endothelial function in a population of overweight but otherwise healthy young individuals. As endothelial dysfunction may contribute to increased risk in CVD development, our finding provides a further rationale to advocate decreasing SF consumption while increasing polyunsaturated and monounsaturated fat in young individuals.

## Author contributions

The authors have contributed to conception: EL, NS, GH, MS, GL, design and conduct of the experiment: EL, SP, NE, CS, MG, GL, and data interpretation: EL, RB, AT, JD, GH, MS, NS, GL.

### Conflict of interest statement

The authors declare that the research was conducted in the absence of any commercial or financial relationships that could be construed as a potential conflict of interest.

## Footnotes

^1^Nutrient Reference Values for Australia and New Zealand. In Department of Health and Ageing NZMoH, Canberra, ACT.

^2^Global Recommendations on Physical Activity for Health. WHO Guidelines Approved by the Guidelines Review Committee, Geneva.

## Abbreviations

SF: Saturated fat
CVD: Cardiovascular disease
CHD: Coronary heart disease
BP: Blood pressure
RHI: Reactive hyperemia index
PAT: Pulse amplitude tonometry
CRP: C-reactive protein
HDL: High-density lipoprotein
LDL: Low-density lipoprotein
BMI: Body mass index
